# Correction: Luo, M. Natural Immunity against HIV-1: Progression of Understanding after Association Studies. *Viruses* 2022, *14*, 1243

**DOI:** 10.3390/v15061323

**Published:** 2023-06-05

**Authors:** Ma Luo

**Affiliations:** 1 National Microbiology Laboratory, Public Health Agency of Canada, Winnipeg, MB R3E 3L5, Canada; ma.luo@phac-aspc.gc.ca or ma.luo@umanitoba.ca; 2 Department of Medical Microbiology and Infectious Diseases, Rady Faculty of Health Sciences, University of Manitoba, Winnipeg, MB R3E 0J9, Canada

The author wishes to make the following corrections to this paper [1]:

Replacement of references to 63, 67, and 68 of this manuscript because these references are incorrect. The incorrect version was accidently introduced during the revision stage. The corrected references are listed below.

“63. Sampathkumar, R.; Peters, H.O.; Mendoza, L.; Bielawny, T.; Ngugi, E.; Kimani, J.; Wachihi, C.; Plummer, F.A.; Luo, M. Influence of HLA class I haplotypes on HIV-1 seroconversion and disease progression in Pumwani sex worker cohort. *PLoS ONE* **2014**, *9*, e101475.

67. Peterson, T.A.; Kimani, J.; Wachihi, C.; Bielawny, T.; Mendoza, L.; Thavaneswaran, S.; Narayansingh, M.J.; Kariri, T.; Liang, B.; Ball, T.B.; et al. HLA class I associations with rates of HIV-1 seroconversion and disease progression in the Pumwani Sex Worker Cohort. *Tissue Antigens* **2013**, *81*, 93–107.

68. Turk, W.J.; Kimani, J.; Bielawny, T.; Wachihi, C.; Ball, T.B.; Plummer, F.A.; Luo, M. Associations of human leukocyte antigen-G with resistance and susceptibility to HIV-1 infection in the Pumwani sex worker cohort. *Aids* **2013**, *27*, 7–15.”.

The author states that the scientific conclusions are unaffected. This correction was approved by the Academic Editor. The original publication has also been updated.

## References

[1] Luo M. (2022). Natural Immunity against HIV-1: Progression of Understanding after Association Studies. Viruses.

